# Pain Experience after Dental Implant Placement Compared to Tooth Extraction

**DOI:** 10.1155/2021/4134932

**Published:** 2021-08-31

**Authors:** Alaa W. AlQutub

**Affiliations:** Oral and Maxillofacial Surgery Department, Um AlQura University, Faculty of Dentistry, Makkah 24225, Saudi Arabia

## Abstract

**Background:**

A patients' main concern when visiting the dentist is the pain experience during the procedure and postoperatively. Patients who are undergoing dental surgical procedures in particular may experience more concerns and higher level of anxiety and stress that can affect their psychology and decision-making ability. A thorough discussion with the patients about the planned surgical procedure and the expected postsurgical pain and discomfort level is crucial to reduce their fear and stress. Despite increasing popularity of dental implants, limited data are available on pain experience related to surgical implant placement. This review is to discuss and compare postoperative pain and discomfort level after dental implant placement procedure and tooth extraction. The review has a clinical significance as it can be used as a reference when explaining to the patients about the anticipated pain and discomfort level after implant placement.

**Conclusion:**

Informing patients about implant placement surgical procedure and the anticipated postsurgical pain can reduce their anxiety level and affect postsurgical pain and discomfort. Implant placement surgical procedure is less unpleasant than tooth extraction, with less postsurgical pain and limitation of daily activities.

## 1. Introduction

Fear of dental treatment has been ranked as the 4th most common fear among the population [1]. The most feared dental procedures in dentistry ranked by the patients are drilling, anesthetic injection, and extraction. Dental fear mostly starts in childhood in patients with previous traumatic dental experiences as the main causative factor. In this case, the dentist's professional behavior plays a major role in creating such a fear. However, for acquired fear in adult years, the main reason is pain. Patients' fear and pain anticipation towards treatment may not consistently reflect their actual experience of treatment as they may expect a stimulus to be more painful than what they actually perceived [2].

To manage and control patients' fear and anxiety before a planned dental surgical procedure, a thorough discussion should be attempted. Providing information to patients can help dentally anxious individuals prepare for treatment and subsequently reduce anxiety levels [3].

As dental implants acquired much popularity as a first treatment option chosen by the patients, limited information is available on the pain experience associated with the surgical placement of dental implants [4]. Comparing this experience to other dental surgical experiences such as dental extraction is more relevant to the patients as it can help them to understand the anticipated pain after implant surgery, hence influencing their decision-making process. Moreover, they should understand that there are some contributing factors that might increase the pain perception after implant placement surgery.

## 2. Effect of Anxiety Level on Pain Experience

Patients tend to overestimate the fear of pain of the dental treatment that they have not experienced, compared to those they have experienced in the past [5]. Most of the candidates for surgical implant placement do not have an experience with implant surgery, and subsequently, they become more anxious about the associated pain which can affect their certainty about the treatment [4].

On the other hand, the majority of these candidates may have an experience of at least one tooth extraction. Comparing the two surgical experiences will help the patients to relate the anticipated pain after implant surgery which can influence their decision-making on proceeding with the treatment providing clear and accurate information about expected pain during treatment which will result in more reliable communication between clinicians and patients. This will enable patients to have a more realistic expectation of the treatment process and reveal all risks that might influence or conflict with their decisions [6].

Well-informed patients about their procedures seem to be less anxious and more comfortable [7].

A qualitative study of patients' views of techniques to reduce dental anxiety was conducted by Wang et al. [3]. Four factors were acknowledged as possible ways to reduce patients' anxiety levels including preparedness, teamwork, reinforced trust, and tailored treatment plan. In this study, patients preferred to be prepared for the treatment by having more information about the steps of the procedure. They also indicated that engaging them in the process of treatment plan formulation and decision-making will reduce their anxiety and build up the trust with their dentists. Importantly, participants wanted to know about the anticipated discomfort during and after treatment, the cost, expected treatment time, and number of appointments needed to complete the treatment [3]. This study supports the concept that well-informed patients will be less anxious about the dental treatment.

Dental anxiety has been reported as having direct influence on pain perception in dental procedures in different laboratory and clinical studies. It is closely linked to painful stimulus and increased pain perception. Therefore anxious patients may experience more pain that lasts longer with exaggerated memory of pain [8].

There is a positive relation between the patient's fear and anxiety level and pain perception after dental procedures. Croog et al. [9] assessed relationships between presurgery psychological characteristics and postsurgery pain response and effect on life activities following each of two sessions of periodontal surgery. Forty-two female periodontal patients with no previous surgical experience were evaluated. They indicated that presurgical scores on dental anxiety, fatigue, and depression were positively associated with measures of postsurgical pain after the first surgery. However, a weaker pattern of associations was evident after the second surgery. The study points to the relevance of presurgical psychological condition as a predictor of postsurgical pain and impairment of life activities. It is indicated that postsurgical pain and discomfort can possibly be reduced by attempts to control dental anxiety and emotional distress prior to surgery. It also supports the perception that patients' anxiety level is reduced with the past dental experience [9].

## 3. Pain Experience after Implant Surgery Compared to Tooth Extraction

There are limited data comparing the pain experience of dental extraction with dental implant surgery on the same patient. This can be advantageous as the pain experience reported by the same subject will be more consistent and the comparison will be more reliable.

One study that compared the pain experience in patients who went through both tooth removal and dental implant surgery was carried out by Tabrizi et al. [10]. They conducted a crossover study on 40 patients and their pain severity at 12, 24, 48, and 72 hours after each procedure was assessed by using a visual analog scale (VAS) as self-reported. Their results showed that the pain of dental implant surgery decreased faster than tooth extraction with time, and that the postsurgical pain with implant surgery is mild with moderate inflammation. They concluded that patients who had experienced both tooth extraction and a dental implant placement reported significantly less pain in implant surgery [10].

This finding is in line with a conclusion of a study conducted by Abolfazil et al. [11]. The study was cross-sectional, and the patients underwent simple tooth extraction for posterior teeth under local anesthesia and received ibuprofen (400 mg) every 6 hours for postsurgical pain management. Two months later, dental implant surgeries were carried out for all patients, and they received ibuprofen (400 mg) every 6 hours as well. Pain level was assessed by VAS immediately after procedure and in the interval time of 6 hours and 1, 3, and 7 days, respectively. They stated that the level of postsurgical pain after tooth extraction was more than implant surgery [11].

Pain and inflammation following the placement of dental implants were assessed by González-Santana et al. [12]. They studied pain and swelling in the first-week postimplant surgical placement reported by the patients using verbal and visual analog scale (VAS). Most of the patients reported slight pain with a peak intensity 6 hours after surgery and moderate swelling with a peak intensity of inflammation after 48 hours. Analysis of pain 6 hours postoperation showed a statistically significant correlation to the number of implants, as increased pain was recorded in the patients subjected to a large number of dental implants. They concluded that pain after implant placement is mild with moderate inflammation [12].

Al-Khabbaz et al. [4] assessed the pain associated with surgical implant placement in a multicenter, prospective study. Patients' mean pain scores were evaluated with 0 to 10 scale during 24 hours and 1, 6, and 12 weeks after surgery. They reported that pain experience following the implants' surgical placement was generally mild and gradually decreased with time [4].

Pain experience and anxiety following dental implant placement was investigated by Hashem et al. [13] using questionnaires by recording pain intensity interference with daily activities on a visual analog scale (VAS). They also analyzed pain and anxiety by collecting salivary cortisol 1 week before surgery, the day of surgery, and 3 and 6 days postoperatively. Their results stated that most patients reported mild to moderate pain and interference with daily activities after implant placement. Average pain experience decreased significantly with time, and limitation of daily activities was highest on the first postsurgical day and diminished to about half the maximum level by the 2nd or 3rd day. Patients scored the highest anxiety level on the day of surgery; however, the salivary cortisol level did not validate this as no difference was presented. It is concluded that implant placement is a mild to moderately painful and anxiety-provoking procedure with some limitations of daily activities and symptoms are expected to occur during the first 3 postsurgical days [13].

A comparison study conducted by Yao et al. [6] revealed that surgical tooth extraction is significantly associated with more pain and bleeding at the first day of healing. However, more swelling and bruising were associated with implant placement with guided bone regeneration procedure. It is reported that healing events of straightforward implants are similar to those of simple extraction [6].

The patients' perspective, i.e., how patients perceive their oral health, is being recognized recently as a significant outcome in modern implant dentistry. A study was conducted to assess how patients perceive the perisurgical stages of implant placement and whether the overall perceived burden of the surgical process is related to specific stage of treatment and to compare patients' perceptions during implant placement with tooth extraction. Considering treatment stages, burdens were highest for anesthesia and lowest for side effects. The study results demonstrated that even though implant placement requires basically similar surgical procedure and instruments for incision, mucoperiosteal flap reflection, bone drilling, and suturing, patients seem to perceive the procedures related to bone and soft tissue less burdensome than during surgical tooth extraction. It can be concluded that implant placement was rated as least burdensome, especially in relation to bone and soft tissue manipulations such as pressure during incision or vibration due to drills compared with surgical tooth extraction [14].

## 4. Factors Affecting Pain Experience in Implant Patients

Many factors are associated with the reported pain intensity in relation to the implant placement procedure such as operator experience, female gender, and surgical difficulty as reported by Al-Khabbaz et al. [4].

The surgeon's experience plays a role in reducing the perisurgical and postsurgical pain [15]. Morin et al. stated that senior surgeons produced significantly less pain than juniors during the implant placement procedure. They also found that pain after implant placement perceived differently based on the patients' gender, with females reporting greater overall pain intensity than males. However, men are more disturbed by the low levels of pain during healing than women [16].

A number of implants placed and the related regenerative procedures (e.g., guided bone regeneration, sinus lift, and split ridge) are associated with increased postsurgical pain and swelling. Moreover, pain experience is more intense in older patients and smokers [12, 13].

It is proven that the longer the duration of the procedure, the more swelling, pain and discomfort perceived by patients [14].

Location of the placed implant and the proximity to vital structures is correlated with pain perception. Swelling is greater in patients with implants placed in the posterior versus anterior zone and in free end spaced or totally edentulous patients versus interdental spaces.

González-Santana et al. [12], who studied the pain and swelling in the first-week postimplant surgical placement reported by the patients using VAS, analyzed the relation between inflammation 48 hours after the operation and the different study variables. Statistically significant correlations were found: more advanced patient age, surgery in edentulous patients and free extremes, and surgical approaches in the posterior area of maxilla and mandibula.

Moreover, a significant association was observed between swelling and more number of implants placed and with surgery involving sinus lift or bone regeneration procedures [12]. The results of Yao et al. [6] also reported that patients who had implant placement with guided bone regeneration procedure may experience more swelling and bruising [6].

Location of the placed implant and the proximity to vital structures is correlated with pain perception. Swelling was reported to be greater in patients with implants placed in the posterior versus anterior zone and in free end spaced or totally edentulous patients versus interdental spaces [12].

Timing of implant placement and loading are also related to the pain experience. Immediate implants may be associated with more discomfort as patients' undergo an extensive surgical procedure of both tooth extraction and implant placement. Urban and Wenzel reported that patients experienced low to moderate pain with severe swelling after immediate implant placement in the molar area with the regenerative procedure [17]. Immediate loading may induce more postoperative pain and swelling than conventional or delayed loading [18].

In terms of flap design, flapless procedure has less pain intensity and duration and faster healing time than open flap procedures. There is a relatively high probability (43%) of experiencing no pain with no medications, and this probability could be increased by taking steroidal anti-inflammatory agents [19]. Khouly et al. reported that short-term use of analgesic medications, in the first 72 hours, is sufficient for postsurgical pain management in dental implant surgery [20]. On the other hand, it is recommended that analgesics should be prescribed during the first week after tooth extraction indicating that tooth extraction has a prolonged unpleasant healing process that necessitates using analgesics up to the 7th postextraction day. [21].

Minimally invasive procedures are always preferred to reduce patients' anxiety and the pain experience and hence increase the treatment acceptance rate by the patient.

## 5. Discussion

It is suggested that pain intensity is higher with tooth extraction compared to the implant placement procedure. This can be explained as most of the extracted teeth are usually chronically inflamed or symptomatic. There is a significant relation between pre-extraction tooth status and the pain intensity perceived after extraction, as postextraction pain can increase 3 folds in patients with symptomatic teeth [22, 23]. The reason for such pain is most probably due to the synergistic effect of different inflammatory mediators, produced by the pre-existing inflammatory condition that produces a long-lasting increase in the nociceptors activity [24]. Therefore, inflamed teeth with triggered nociceptors and carious teeth with excited nerve endings will produce more pain when extracted as compared with noninflamed teeth [21]. Subsequently, it is expected that implant placement procedure that does not involve dealing with inflamed teeth is more pleasant and has less postsurgical pain perception.

Another explanation is that dental implant surgery is a more controllable and less traumatic procedure than tooth extraction. Subsequently, less pain and discomfort is expected postsurgically.

More research studies should be carried out to compare between surgical implant placement and other oral surgical procedures on the same individuals with larger sample sizes, taking into consideration the contributing factors that might affect the pain perception by implant candidates.

## 6. Conclusion

Informing patients about the anticipated surgical procedure of implant placement and the postsurgical pain intensity can reduce their anxiety level and subsequent postsurgical pain and discomfort. Patients can be informed that, in general, implant placement surgical experience is less unpleasant compared to tooth extraction with less postsurgical pain and limitation of daily activities. However, some factors can increase the pain intensity and discomfort level on individual bases.

## Data Availability

The data supporting this review are from previously reported studies and datasets, which have been cited.

## Abbreviations

VAS: Visual Analog Scale.

## References

[1] Tanidir A. N., Atac M. S., Karacelebi E. (2016). Information given by multimedia: influence on anxiety about extraction of impacted wıidom teeth. *British Journal of Oral and Maxillofacial Surgery*.

[2] Klages U., Ulusoy O., Kianifard S., Wehrbein H. (2004). Dental trait anxiety and pain sensitivity as predictors of expected and experienced pain in stressful dental procedures. *European Journal of Oral Sciences*.

[3] Wang M. C., Vinall-Collier K., Csikar J., Douglas G. (2017). A qualitative study of patients’ views of techniques to reduce dental anxiety. *Journal of Dentistry*.

[4] Al-Khabbaz A. K., Griffin T. J., Al-Shammari K. F. (2007). Assessment of pain associated with the surgical placement of dental implants. *Journal of Periodontology*.

[5] Van Wijk A. J., Hoogstraten J. (2005). Experience with dental pain and fear of dental pain. *Journal of Dental Research*.

[6] Yao J., Lee K., McGrath C., Wu Y. N, Li K. Y., Mattheos N. (2016). Comparison of patient-centered outcomes after routine implant placement, teeth extraction, and periodontal surgical procedures. *Clinical Oral Implants Research*.

[7] Berggren U., Meynert G. (1984). Dental fear and avoidance: causes, symptoms, and consequences. *Journal of the American Dental Association*.

[8] Wong M., Kaloupek D.-G. (1986). Coping with dental treatment: the potential impact of situational demands. *Journal of Behavioral Medicine*.

[9] Croog S. H., Baume R. M., Nalbandian J. (1995). Pre-surgery psychological characteristics, pain response, and activities impairment in female patients with repeated periodontal surgery. *Journal of Psychosomatic Research*.

[10] Tabrizi R., Mohajerani H., Nabtieh A., Shafiei S. (2020). Do patients have the same experience of pain following tooth extraction and dental implants?. *Annals of maxillofacial surgery*.

[11] Abolfazli S., Gravand E., Hedayatian M., Rohani A., Shakerian K. (2019). Comparison of pain between tooth extraction and implant surgery. *Journal of Research in Medical and Dental Science*.

[12] González-Santana H., Peñarrocha-Diago M., Guarinos-Carbó J., Balaguer-Martínez J. (2005). Pain and inflammation in 41 patients following the placement of 131 dental implants. *Medicina Oral, Patologia Oral Cirugia Bucal*.

[13] Hashem A. A., Claffey N. M., O’Connell B. (2006). Pain and anxiety following the placement of dental implants. *The International Journal of Oral & Maxillofacial Implants*.

[14] Reissmann D., Poulopoulos G., Durham J. (2015). Patient perceived burden of implant placement compared to surgical tooth removal and apicectomy. *Journal of Dentistry*.

[15] Eli I., Schwartz-Arad D., Baht R., Ben-Tuvim H. (2003). Effect of anxiety on the experience of pain in implant insertion. *Clinical Oral Implants Research*.

[16] Morin C., Lund J. P., Villarroel T., Clokie C. M. L., Feine J. S. (2000). Differences between the sexes in post-surgical pain. *Pain*.

[17] Urban T., Wenzel A. (2010). Discomfort experienced after immediate implant placement associated with three different regenerative techniques. *Clinical Oral Implants Research*.

[18] Mundt T., Passia N., Att W. (2017). Pain and discomfort following immediate and delayed loading by over dentures in the single mandibular implant study (SMIS). *Clinical Oral Investigations*.

[19] Fortin T., Bosson J. L., Isidori M., Blanchet E. (2006). Effect of flapless surgery on pain experienced in implant placement using an image-guided system. *The International Journal of Oral & Maxillofacial Implants*.

[20] Khouly I., Braun R. S., Ordway M. (2021). Post-operative pain management in dental implant surgery (2021) a systematic review and meta-analysis of randomized clinical trials. *Clinical Oral Investigations*.

[21] Al-Khateeb T. H., Alnahar A. (2008). Pain experience after simple tooth extraction. *Journal of Oral and Maxillofacial Surgery*.

[22] Ruta D. A., Bissias E., Ogston S., Ogden G. R. (2000). Assessing health outcomes after extraction of third molars: the postoperative symptom severity (PoSSe) scale. *British Journal of Oral and Maxillofacial Surgery*.

[23] Slade G. D., Foy S. P., Shugars D. A., Phillips C., White R. P. (2004). The impact of third molar symptoms, pain, and swelling on oral health-related quality of life. *Journal of Oral and Maxillofacial Surgery*.

[24] Levine J. (1984). Pain and analgesia: the outlook for more rational treatment. *Annals of Internal Medicine*.

